# What is Minimally Adequate Treatment of Psychosis and Should Duration of Inadequate Treatment be a Clinical and Research Target? A Perspective and State-of-the-Art Review

**DOI:** 10.1093/schbul/sbag017

**Published:** 2026-03-21

**Authors:** Martin Osugo, Oliver D Howes

**Affiliations:** Department of Psychosis Studies, Institute of Psychiatry, Psychology & Neuroscience, King’s College London, London, SE5 8AB, United Kingdom; Psychiatric Imaging Group, Medical Research Council, London Institute of Medical Sciences, Hammersmith Hospital, London, W12 0HS, United Kingdom; South London and Maudsley NHS Foundation Trust, London, SE5 8AZ, United Kingdom; Department of Psychosis Studies, Institute of Psychiatry, Psychology & Neuroscience, King’s College London, London, SE5 8AB, United Kingdom; Psychiatric Imaging Group, Medical Research Council, London Institute of Medical Sciences, Hammersmith Hospital, London, W12 0HS, United Kingdom; South London and Maudsley NHS Foundation Trust, London, SE5 8AZ, United Kingdom

**Keywords:** antipsychotics, inadequate treatment, DUP, service evaluation, outcomes, guidelines, FEP, chronic psychosis, maintenance treatment

## Abstract

**Background and Hypothesis:**

Persistent symptoms and disability are common in psychotic disorders. This may be partly attributable to inadequate antipsychotic treatment, but there has not been a recent overview of what constitutes inadequate treatment and its impact on outcomes.

**Study Design:**

We focus on the latest meta-analyses to critically appraise the relationship between markers of inadequate antipsychotic treatment and outcomes from the first episode of psychosis onwards, relating outcomes to periods without antipsychotic use, antipsychotic treatment of subtherapeutic dose/duration, and antipsychotic partial/non-adherence.

**Study Results:**

Inadequate antipsychotic treatment is common (non-adherence rates = 44%-56%), and repeatedly associated with poorer outcomes across several key patient-centered outcomes, including increased risk of relapse (relative risk (RR) up to 2.70, *n* = 13 988), more severe overall symptoms (standardized mean difference (SMD) up to 0.78, *n* = 8878), poorer quality-of-life (SMD up to 0.50, *n* = 1421), poorer functioning (SMD up to 0.55, *n* = 1988) and higher mortality (RR up to 1.83, *n* = 272 030). We also find there is more evidence for schizophrenia than other psychotic disorders.

**Conclusions:**

We identify that there are no operationalized criteria for the minimally adequate treatment of psychosis, in contrast to major depression, for example. We propose that a longer duration of inadequate treatment (DIT) may be an important predictor of outcome, although this has not been tested. To address this and support the development of interventions to reduce inadequate treatment, we propose operationalized criteria for the minimally adequate treatment of psychosis and the DIT, proposing both clinically applicable and research criteria. Finally, we consider future directions for research and practice.

## Introduction

There is a substantial body of evidence that antipsychotic treatment improves outcomes in psychotic disorders.^1-4^ For example, a systematic review and meta-analysis of all double-blind randomized controlled trials (RCTs) conducted from 1953-2016 comparing antipsychotic treatment to placebo in acute exacerbations of schizophrenia (*n* > 28 000) found that antipsychotic treatment was superior to placebo in the treatment of positive, negative, depressive, and overall symptoms.^1^ Moreover, antipsychotic treatment was also superior to placebo for key patient-centered outcomes such as quality-of-life and functional status^1^. Updated network meta-analysis of double-blind RCTs, including over 53 000 patients with acute exacerbations from short-term studies (3-12 weeks) and over 13 500 patients with stable schizophrenia from long-term studies (6-12 months), have extended these findings to show that the vast majority of the 32 antipsychotics tested are individually superior to placebo for symptomatic and functional outcomes in both acute and long-term studies, and for relapse prevention over 6-12 months.^2^^,^^3^

Nevertheless, incomplete recovery, persistent symptoms, and disability are common in psychotic disorders, and there have been few advances in drug treatment over the past 40 years.^5-7^ This has led to increasing interest in other modifiable factors that may determine outcomes.^6^ One such factor that has been widely investigated is the duration of untreated psychosis (DUP), most commonly defined as the period from the onset of psychotic symptoms to the initiation of antipsychotic treatment.^6^ An umbrella review of multiple meta-analyses comprising a total of 129 observational studies and including over 25 000 people with psychosis assessed the relationship between DUP and various outcomes.^6^ This found that a longer DUP was associated with poor outcomes at long-term follow-up, including more severe positive, negative, and overall symptoms, a lower chance of remission, poorer functional outcome, and worse quality-of-life.^6^ Although there is debate as to whether this relationship is causal, findings such as these have nevertheless influenced mental health services worldwide to implement early intervention models, which have the reduction of DUP as one of their primary aims.^8^^,^^9^

**Figure 1:**
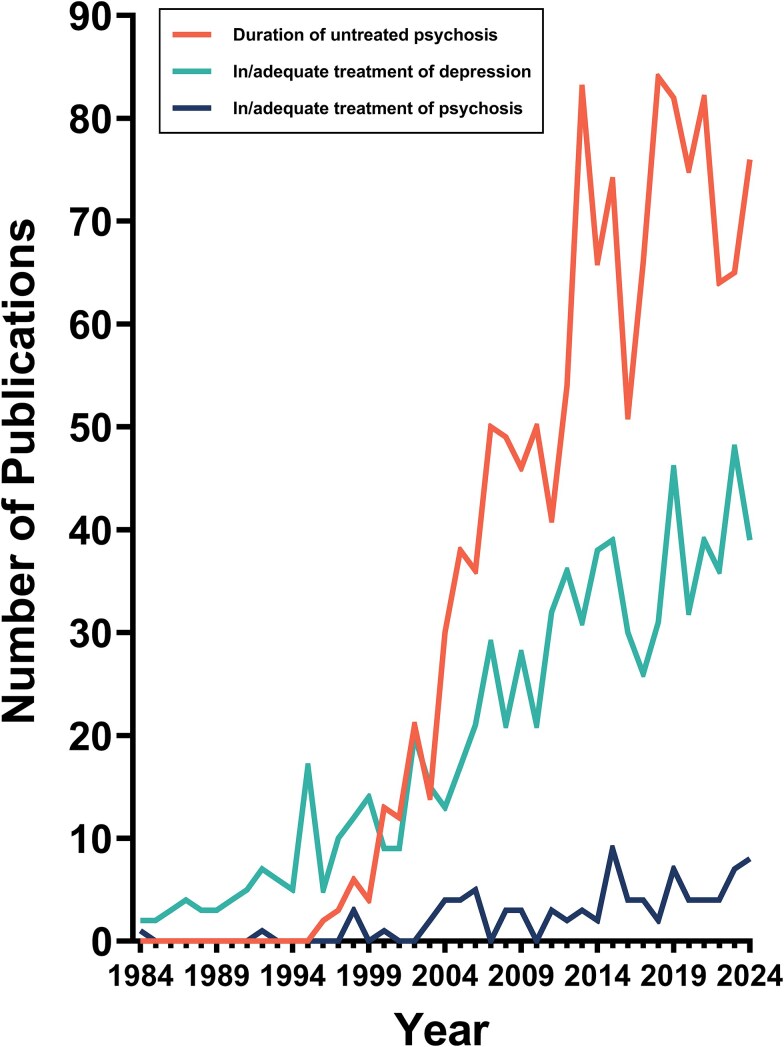
Number of publications per year in PubMed for “duration of untreated psychosis” compared to the number of publications per year in PubMed for a) “in/adequate treatment” and “psychosis,” b) “in/adequate treatment” and “depression”

The evidence that antipsychotics improve outcomes, and that longer delay to first antipsychotic treatment is associated with poorer outcomes, raises the question of whether subsequent periods without adequate antipsychotic treatment also influence outcomes throughout the course of the illness. To assess the extent of research into this over the past 40 years, we conducted a search of PubMed on June 5, 2025, using the terms “duration of untreated psychosis”, and (“adequate treatment” or “inadequate treatment”) and “psychosis.” We also included the terms “duration of untreated depression”, and (“adequate treatment” or “inadequate treatment”) and “depression,” as a comparator. The results are presented in Figure 1, showing increasing research interest in DUP, particularly in the last 15 years, comparatively lower but still substantial research into the adequate treatment of depression, and minimal research into adequate treatment of psychosis. There was also minimal research into duration of untreated depression (data not shown), demonstrating that the focus of depression research has been on adequate treatment irrespective of illness stage, in contrast to psychosis research, which has focused on early intervention and not on the adequate treatment of people with chronic illnesses.

This lack of research interest may be partly explained by a lack of widely used criteria for what constitutes minimally adequate treatment of psychosis. Such criteria exist for major depressive disorder, and there are prior consensus criteria on the minimum treatment required for establishing treatment resistance in schizophrenia.^10^^,^^11^ These guidelines considered that treatment of schizophrenia may be considered inadequate because either (1) no antipsychotic has been prescribed, or (2) the antipsychotic is not prescribed at a therapeutic dose or duration, or (3) the antipsychotic is not taken as prescribed.^11^ To evaluate the importance of inadequate treatment of psychosis, we review evidence for a relationship between inadequate treatment and outcomes from the first-episode of psychosis (FEP) onwards, focusing on the latest meta-analyses. We review the evidence for each of these factors in turn, before considering their implications for research and clinical services, and considering how measurement of minimally adequate treatment and duration of inadequate treatment (DIT) could be operationalized.

## Methods

We identified relevant articles by searching PubMed for combinations of these measures of inadequate antipsychotic treatment (eg, “antipsychotic” and “non-compliance” or “dose–response” or “LAI,” etc.) and outcomes (eg, “outcomes” or “quality-of-life” or “relapse” or” functioning”, etc.), supplemented by screening the reference lists of relevant articles and the authors’ knowledge. We considered FEP and chronic psychosis separately, as it has been proposed that treatment response may differ in these groups, and they are often managed in different services.^6^^,^^12^

Duration of untreated psychosis studies were not included as they test the relationship between delays in first treatment initiation and outcomes in the first episode only, whereas we aimed to understand whether inadequate antipsychotic treatment after this point (ie, throughout the entire illness course) also predicts outcome. Where there were multiple meta-analyses on a measure/outcome relationship, we preferred the latest meta-analysis based on data from double-blind RCTs. If no such meta-analysis was available, we then sequentially considered meta-analyses of single-blind/open-label RCTs, meta-analyses of observational studies, and finally individual studies (preferring recent articles and larger sample sizes).

### Do Periods of No Antipsychotic Treatment Influence Outcomes?

Meta-analysis of longitudinal studies including over 270 000 people with chronic psychosis shows that antipsychotic non-users have approximately double the long-term mortality rate compared to antipsychotic users^13^ (Table 1). Contrastingly, a Danish national database study found that people who discontinued antipsychotic treatment and sustained this following a first-episode of schizophrenia had better outcomes in terms of family functioning and employment status at long-term follow-up compared to people who used antipsychotics continuously.^14^ These observational findings should be interpreted cautiously, as they may be explained by systematic differences between antipsychotic users and non-users, for example, in illness severity or health behaviors. Some observational studies have addressed this limitation; in a Finnish national database study where the analysis was conducted longitudinally within the same individuals, periods of antipsychotic non-use following the first-episode of schizophrenia were associated with an almost three times greater risk of first relapse compared to periods with antipsychotic prescription at standard doses.^15^ This study also observed a similar relationship between periods of antipsychotic non-use and relapse for subsequent relapses throughout the course of the illness.^15^ These findings were extended by a combined analysis of Finnish and Swedish national cohort studies, which found that each individual antipsychotic was superior to antipsychotic non-use for preventing relapse in people with stable schizophrenia spectrum disorders.^16^

**Table 1:** Summary of Meta-Analytic Evidence on Relationships Between Markers of Inadequate Treatment and Outcome

**Outcome**	**Measure of inadequate treatment**	**Population**	**Study design**	**Duration**	**Outcome (k, *n*)**	**Association between inadequate treatment and outcome**	**Effect size; [95% CI], *P*-value**

**Relapse**	No antipsychotic treatment (antipsychotic continuation vs withdrawal)^17^	Remitted FEP, stabilized on antipsychotic therapy	Meta-analysis of RCTs (60% double-blind)	12-24 months (mean 19 months)	k = 10, *n* = 776	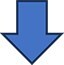	12 months: RR = 2.13; [1.61-2.86], *P* < .00001 18-24 months: RR = 1.75; [1.25-2.44], *P* = .001

	No antipsychotic treatment (antipsychotic continuation vs withdrawal)^18^	Schizophrenia spectrum disorders, stabilized on antipsychotic therapy	Network meta-analysis of RCTs (80% double-blind)	≥6 weeks	k = 98, *n* = 13 988	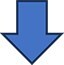	RR = 2.70; [2.33-3.13], *P* = nr

	Inadequate dose (dose maintenance vs dose reduction)^18^	Schizophrenia spectrum disorders, stabilized on antipsychotic therapy	Network meta-analysis of RCTs (80% double-blind)	≥6 weeks	k = 98, *n* = 13 988	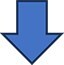	RR = 1.82; [1.41-2.38], *P* = nr

	Inadequate dose (standard vs very low dose)^19^	Stable schizophrenia or schizoaffective disorder	Meta-analysis of double blind RCTs	≥24 weeks	k = 13, *n* = 3058	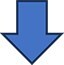	RR = 1.72; [1.29-2.29], *P* = .0002

	Inadequate dose (standard vs low dose)^19^				k = 11, *n* = 1476	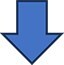	RR = 1.38; [1.04-1.84], *P* = .025

	Poor adherence^20^	FEP	Meta-analysis of prospective longitudinal studies	≥12 months	k = 7, *n* = 577	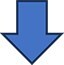	OR = 4.09; [2.55-6.56], *P* < .01

	Poor adherence (continuous treatment vs intermittent treatment)^21^	Chronic schizophrenia, stabilized on antipsychotic treatment	Meta-analysis of RCTs	≥6 months	k = 10, *n* = 1230	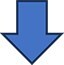	OR = 3.36; [2.36-5.45], *P* < .0001

	Poor adherence (LAI vs oral)^22^	Schizophrenia spectrum disorders	Meta-analysis of RCTs	≥6 months	k = 29, *n* = 7833	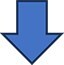	RR = 1.14; [1.01-1.27], *p* = .033

			Meta-analysis of cohort studies	≥6 months	k = 44, *n* = 106 136	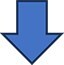	RR = 1.09; [1.02-1.14], *P* = .0044
			Meta-analysis of mirror image studies	≥12 months	k = 28, *n* = 17 876	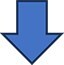	RR = 2.27; [1.96-2.56], *P* < .0001
**Global psychopathology**	No antipsychotic treatment (antipsychotic continuation vs withdrawal)^18^	Schizophrenia spectrum disorders, stabilized on antipsychotic therapy	Network meta-analysis of RCTs (80% double-blind)	≥6 weeks	k = 47; *n* = 8878	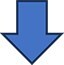	SMD = 0.78; [0.57-0.99], *P* = nr

	Inadequate dose (dose maintenance vs dose reduction)^18^	Schizophrenia spectrum disorders, stabilized on antipsychotic therapy	Network meta-analysis of RCTs (80% double-blind)	≥6 weeks	k = 47; *n* = 8878	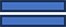	SMD = 0.10; [-0.22-0.43], *P* = nr

	Inadequate dose (Minimum effective dose (MED) vs 2x MED^23^	Acute exacerbation of schizophrenia/schizoaffective disorder	Meta-analysis of double-blind RCTs	4-26 weeks (median 6 weeks)	k = 18, *n* = 3918	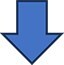	SMD = 0.08; [0.02-0.15], *P* = .01

	Inadequate dose (Minimum effective dose (MED) vs 3x MED^23^				k = 8, *n* = 1363	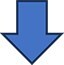	SMD = 0.17; [0.02-0.33], *P* = .03

	Inadequate dose (standard vs very low dose)^19^	Stable schizophrenia or schizoaffective disorder	Meta-analysis of double blind RCTs	≥24 weeks	k = 5, *n* = 1422	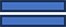	SMD = 0.25; [-0.01-0.52], *P* = .064

	Inadequate dose (standard vs low dose)^19^				k = 3, *n* = 885	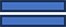	SMD = 0.16; [0.00-0.32], *P* = .050

	Poor adherence (LAI vs oral)^22^	Schizophrenia spectrum disorders	Meta-analysis of RCTs	≥6 months	k = 13, *n* = 4848	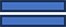	SMD = 0.06; [-0.05-0.17], *P* = .31

			Meta-analysis of cohort studies	≥6 months	k = 5, *n* = 2331	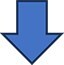	SMD = 0.30; [0.17-0.43], *P* < ·0001
**Positive symptoms**	No antipsychotic treatment (antipsychotic continuation vs withdrawal)^17^	Remitted FEP, stabilized on antipsychotic therapy	Meta-analysis of RCTs	12-24 months	k = 2, *n* = 175	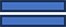	SMD = 0.12; [-0.20-0.43], *P* = .47

	Inadequate dose (Minimum effective dose (MED) vs 2x MED^23^	Acute exacerbation of schizophrenia/schizoaffective disorder	Meta-analysis of double-blind RCTs	4-26 weeks (median 6 weeks)	k = 15, *n* = 3541	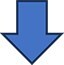	SMD = 0.12; [0.05-0.19], *P* = .001

	Inadequate dose (Minimum effective dose (MED) vs 3x MED^23^				k = 8, *n* = 1363	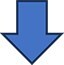	SMD = 0.20; [0.07-0.33], *P* = .003

	Poor adherence (LAI vs oral)^22^	Schizophrenia spectrum disorders	Meta-analysis of RCTs	≥6 months	k = 11, *n* = 3379	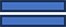	SMD = 0.04; [-0.12-0.19], *P* = .64

**Negative symptoms**	No antipsychotic treatment (antipsychotic continuation vs withdrawal)^17^	Remitted FEP, stabilized on antipsychotic therapy	Meta-analysis of RCTs	12-24 months	k = 2, *n* = 175	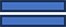	SMD = 0.20; [-0.73-1.12], *P* = .68

	Inadequate dose (Minimum effective dose (MED) vs 2x MED^23^	Acute exacerbation of schizophrenia/schizoaffective disorder	Meta-analysis of double-blind RCTs	4-26 weeks (median 6 weeks)	k = 15, *n* = 3541	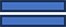	SMD = 0.02; [-0.05-0.08], *P* = .58

	Inadequate dose (Minimum effective dose (MED) vs 3x MED^23^				k = 8, *n* = 1363	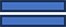	SMD = 0.07; [-0.05-0.19], *P* = .23

	Poor adherence (LAI vs oral)^22^	Schizophrenia spectrum disorders	Meta-analysis of RCTs	≥6 months	k = 10, *n* = 3332	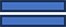	SMD = -0.01; [-0.12-0.11], *P* = .90
**Functional status**	No antipsychotic treatment (antipsychotic continuation vs withdrawal)^18^	Schizophrenia spectrum disorders, stabilized on antipsychotic therapy	Network meta-analysis of RCTs (80% double-blind)	≥6 weeks	k = 13, *n* = 1988	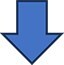	SMD = 0.55; [0.20-0.90], *P* = nr

	Inadequate dose (dose maintenance vs dose reduction)^18^	Schizophrenia spectrum disorders, stabilized on antipsychotic therapy	Network meta-analysis of RCTs (80% double-blind)	≥6 weeks	k = 13, *n* = 1988	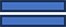	SMD = -0.24; [-0.86-0.39], *P* = nr

	Poor adherence (LAI vs oral)^24^	Schizophrenia or schizoaffective disorder	Meta-analysis of RCTs (20% double-blind)	12-130 weeks	k = 10, *n* = 3540	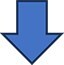	SMD = 0.16; [0.01-0.31], *P* = .04

	Poor adherence (LAI vs oral)^22^	Schizophrenia spectrum disorders	Meta-analysis of cohort studies	≥6 months	k = 2, *n* = 1133	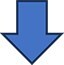	SMD = 0.54; [0.02-1.06], *P* = .043

**Quality-of-life**	No antipsychotic treatment (antipsychotic continuation vs withdrawal)^17^	Remitted FEP, stabilized on antipsychotic therapy	Meta-analysis of RCTs	12-24 months	k = 2, *n* = 175	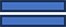	SMD = 0.13; [-0.19-0.44], *P* = .44

	No antipsychotic treatment (antipsychotic continuation vs withdrawal)^18^	Schizophrenia spectrum disorders, stabilized on antipsychotic therapy	Network meta-analysis of RCTs (80% double-blind)	≥6 weeks	k = 8, *n* = 1421	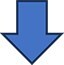	SMD = 0.50; [0.15-0.85], *P* = nr

	Inadequate dose (dose maintenance vs dose reduction)^18^	Schizophrenia spectrum disorders, stabilized on antipsychotic therapy	Network meta-analysis of RCTs (80% double-blind)	≥6 weeks	k = 8, *n* = 1421	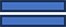	SMD = 0.10; [-0.46-0.66], *P* = nr
**Mortality**	No antipsychotic treatment (antipsychotic use vs non-use)^13^	Schizophrenia spectrum disorders, up to 30% affective psychosis	Meta-analysis of longitudinal cohort studies	3-14 years	k = 7, *n* = 272 030	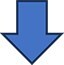	RR = 1.83; [1.61-2.08], *P* = nr

	Poor adherence (LAI vs oral)^22^	Schizophrenia spectrum disorders	Meta-analysis of RCTs	≥6 months	k = 14, *n* = 5676	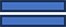	RR = 1.25; [0.66-2.38], *P* = .49

	Meta-analysis of cohort studies			≥6 months	k = 3, *n* = 22 697	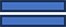	RR = 1.23; [0.88-1.72], *P* = .22

Effect sizes are expressed such that an OR/RR greater than 1, or positive SMD represents poorer outcome with inadequate treatment. Abbreviations: FEP = first-episode of psychosis, LAI = long-acting injectable, nr = not reported, OR = odds ratio, RR = relative risk, RCTs = randomized controlled trial, SMD = standardized mean difference

Nevertheless, some people find the adverse effects of antipsychotics distressing, disabling, or intolerable, and some antipsychotics may have effects on gray matter volumes, inspiring trials to assess the impacts of stopping antipsychotic treatment.^25–31^ In patients with remitted FEP stabilized on antipsychotic therapy, meta-analysis of RCTs (60% double-blind) demonstrates that antipsychotic discontinuation is associated with approximately double the rate of relapse over 1 and 2-year follow-up compared to maintenance treatment, although symptomatic outcomes and quality-of-life do not differ between groups^17^^,^^32^ (Table 1). The increased risk of relapse was not explained by the discontinuation protocol, as this was found both in studies where antipsychotics were discontinued abruptly (k = 4 studies, *n* = 134, relative risk (RR) = 0.40), and when gradually tapered over several months (k = 6, *n* = 605, RR = 0.48).^17^ Similar experimental studies conducted in stable schizophrenia spectrum disorders have assessed the impact of withdrawing antipsychotic treatment on a wider range of outcomes. In this population, meta-analysis of RCTs (80% double-blind) shows that complete withdrawal of antipsychotic treatment and replacement by placebo is associated with poorer overall functioning, more severe overall symptoms, poorer quality-of-life, and greater risk of relapse/hospitalization in comparison to continuous antipsychotic treatment^18^^,^^21^ (Table 1).

Overall, both the observational and experimental studies provide clear evidence that periods of no antipsychotic treatment are associated with poor outcomes at the group level, whilst also showing that some people with psychotic disorders can achieve good outcomes without antipsychotic treatment.^14^^,^^33^^,^^34^

### Does Dose Matter?

Meta-analyses of fixed-dose, double-blind RCTs in acute schizophrenia have identified the minimum effective dose (MED) of antipsychotics for overall symptom reduction in this population, defined as the lowest dose that is consistently more efficacious than placebo.^35^ This analysis found that the MED is 250 mg/day chlorpromazine-equivalent dose (2 mg/day risperidone-equivalent dose).^35^

**Figure 2:**
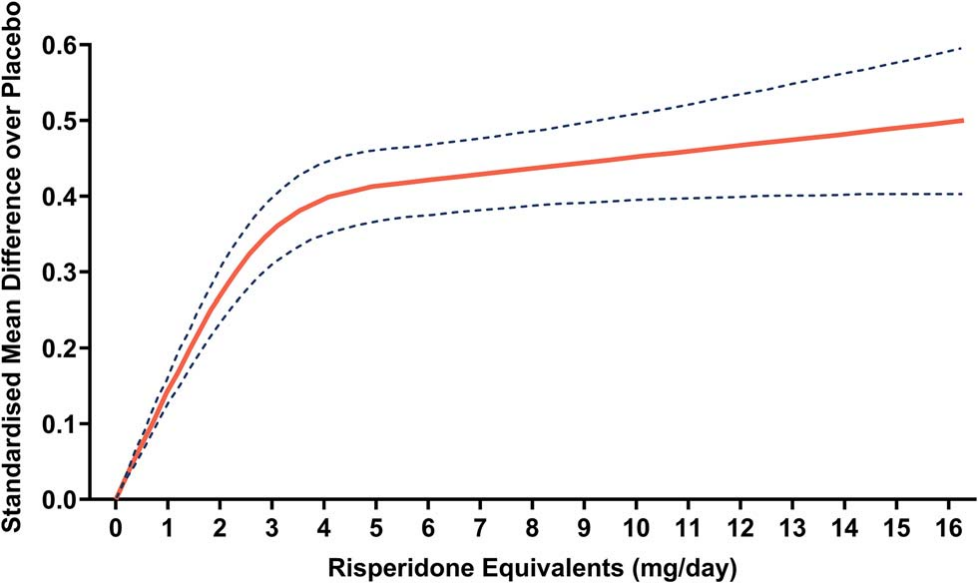
Dose–response curve for total symptoms across antipsychotic drugs, with doses converted to risperidone-equivalent daily dose.^36^ Dose–response curve for total symptoms across 20 pooled antipsychotic drugs (solid line), with doses converted to risperidone equivalents. The 95% CI of the dose–response curve is represented by the dashed lines. Data were extracted from Leucht et al.^36^ using WebPlot digitizer (version 5.2, Automeris LLC; California, United States) and plotted in GraphPad Prism (version 10.2.3, GraphPad software; Massachusetts, United States).

In acute exacerbations of schizophrenia, meta-analysis of placebo-controlled, fixed-dose RCTs (k = 68, 99% double-blind, *n* = 22 495, duration 4-26 weeks) demonstrates a hyperbolic dose–response relationship between total symptom improvement and dose (expressed as daily risperidone-equivalent dose) of 20 pooled antipsychotics. Strikingly, quadrupling the dose from 1 mg to 4 mg/day (expressed as risperidone-equivalent dose) led to a quadrupling of efficacy from a standardized mean difference (SMD) over placebo of 0.1 to 0.4 (Figure 2).^36^ The dose–response curve begins to flatten between approximately 3-5 mg risperidone-equivalent daily, with no significant increase in efficacy seen beyond this.^36^ The dose that produced 50% of the maximum response (ED50) was found to be the same as the minimum effective dose, 2 mg risperidone-equivalent per day.^36^

This meta-analysis also found that individual antipsychotics all had linear dose–response relationships with total symptom improvement at low doses, although individual curves were either hyperbolic or bell-shaped as doses increased. Bell-shaped dose–response relationships for some individual antipsychotics against total symptoms are likely explained by antipsychotic induced negative/extrapyramidal symptoms at higher doses, increasing ratings in these domains, without further benefits against psychotic symptoms.^25^^,^^36^ These findings have been extended by meta-analyses showing that efficacy also increases linearly at low doses for individual antipsychotics against positive and negative symptoms separately in acute schizophrenia (k = 40 double-blind RCTs, *n* = 14 689).^37^ When dose is discretized, findings from meta-analyses of double-blind RCTs in acute schizophrenia are largely consistent with the above meta-analyses where dose is analyzed as a continuous variable; doses two and three times the minimum effective dose (2 mg risperidone-equivalent daily) were found to be more efficacious against total and positive symptoms than the minimum effective doses, although there were no significant differences for negative symptoms^23^ (Table 1).

Other analyses have examined the effect of antipsychotic dose on outcomes in long-term studies of people with chronic schizophrenia who were clinically stable at trial entry.^38^ Based on meta-analyses of fixed-dose studies (k = 26, 92% double-blind, *n* = 4776, duration 6 months-3 years) in this population, the dose–response curve of 13 pooled antipsychotics was again hyperbolic for the relationship between dose and total symptom improvement, as well as the risk of relapse and hospitalization.^38^ A relative reduction in relapse rates of 30% was seen with doses of 2.5 mg risperidone-equivalent daily compared to 0.5 mg daily, but little to no benefit was seen for any outcome as doses were increased beyond risperidone 5 mg daily.^38^ Another way to examine this is to categorize doses into categories. A meta-analysis using this approach in double-blind studies of chronic, stable schizophrenia found that very low doses (1%-49% of standard doses) and low doses (50%-99% of standard doses) were associated with a 72% and 38% increased risk of relapse compared to standard doses, respectively.^19^ In addition, low doses were superior to very low doses for global psychopathology, but not relapse risk^19^ (Table 1). All three dose groups were defined relative to the lower limit of the recommended target dose range for the acute treatment of schizophrenia proposed in the International Consensus Study of Antipsychotic Dosing.^39^ This was done for each antipsychotic individually (eg, 4 mg/day for risperidone, 10 mg/day for olanzapine).^39^ The finding that low doses increase the risk of relapse compared to dose maintenance in stable schizophrenia was confirmed in another meta-analysis using a lower threshold to define low doses (daily chlorpromazine-equivalent doses ≤200 mg/day, approximately 1.6 mg risperidone-equivalent daily).^35^^,^^40^ In addition to this, there is evidence for a gradient of effects on symptomatic and functional outcomes (including relapse) in chronic psychosis from dose maintenance, to dose reduction to treatment discontinuation, based on network meta-analysis of RCTs (80% double-blind) of people with schizophrenia spectrum disorders stabilized on antipsychotic treatment at trial entry^18^ (Table 1).

There is, thus, a substantial body of evidence showing that antipsychotic dose does matter, and that there are mean minimum effective doses for both acute response and long-term maintenance, below which the dose is sub-therapeutic.

### Dose Adherence Matter?

Meta-analysis of longitudinal observational studies in FEP shows that poor adherence is related to a four-fold greater risk of relapse over long-term follow-up^20^ (Table 1). Subsequent to this, it has been shown that even mild non-adherence (defined as non-compliance with 25%-50% of the prescribed dose for at least two consecutive weeks) is associated with increased risk of recurrence of psychotic symptoms (hazard ratio = 5.8).^41^ However, there is little robust evidence from observational studies on the relationship between poor adherence and other outcomes, in part because reliable measures of adherence have not been widely adopted.^42^ However, the impact of partial/non-adherence in terms of the consistency of treatment has, to some degree, been assessed in controlled studies where intermittent treatment or discontinuation has been experimentally tested.

#### Intermittent Treatment Studies

Experimental studies have been conducted where antipsychotic dose reduction/withdrawal has been combined with recommencement of antipsychotic treatment where symptoms recur; a strategy known as intermittent or targeted treatment.^21^ These experimental manipulations mirror some patterns of partial/non-adherence seen in clinical practice, such as where patients reduce or stop treatment when they feel well and restart when symptoms recur.^43^ In people with chronic schizophrenia stabilized on antipsychotic treatment, meta-analysis of RCTs finds that intermittent treatment strategies are associated with a three times increased risk of hospitalization compared to continuous treatment^21^ (Table 1). A recent large open-label RCT (*n* = 253) extended these findings by including people with other stable chronic psychoses in addition to schizophrenia, demonstrating double the risk of relapse following gradual, flexible antipsychotic dose reduction compared to maintenance treatment at 2-year follow-up, although it did not find differences between the groups in terms of self-reported social functioning.^33^

#### Studies of Assured Adherence

A comparison of outcomes between long-acting injectable (LAI) and oral antipsychotic formulations can be considered a proxy measure of the effects of non-adherence, as non-adherence to oral antipsychotics is between 40% and 60%, whilst non-adherence to LAIs is less prevalent and immediately apparent. Real-world evidence shows people on LAIs spend more than twice as many days treated and are 89% more likely to be adherent.^22^^,^^44–46^

Perhaps surprisingly, considering this, in meta-analyses of RCTs, there are no differences between pooled LAIs and pooled oral antipsychotics on symptomatic outcomes, including total and psychotic symptom severity, mortality, or suicide, although meta-analysis of RCTs shows that LAIs are superior to oral antipsychotics in improving functional outcomes and reducing relapse risk^22^^,^^24^ (Table 1). However, although RCTs are the gold standard for efficacy relative to placebo of the same formulation in evidence-based medicine generally, they have significant limitations in comparing outcomes between LAI and oral formulations in psychotic disorders. In this context, the strict inclusion criteria and monitoring in excess of that seen in routine clinical practice select for patients who are likely to adhere to oral medications, resulting in limited generalisability to most clinical settings.^47^^,^^48^ This probably leads to underestimation of the benefits of LAIs in clinical practice, as the effects of assuring adherence with LAIs are limited by ceiling effects. Supporting this conclusion, rates of adherence are similar between LAIs and oral treatments in RCTs, in contrast to real-world evidence discussed above showing considerably greater adherence for patients on LAIs.^22^^,^^46^^,^^49^ Furthermore, RCTs with a more pragmatic (versus experimental) design are significantly more likely to demonstrate a benefit of LAIs versus oral treatment.^50^ To address this, the most up-to-date meta-analysis (k = 137, *n* = 397 319) examined LAI versus oral treatment across study designs.^22^ Consistent with the above, although LAIs reduced relapse compared to oral treatment across all designs, the benefits were greater in mirror-image studies than in RCTs or cohort studies^22^ (Table 1). Moreover, in contrast to findings from RCTs, LAIs were also superior to oral antipsychotics against global psychopathology in cohort studies, and for service utilization in cohort and mirror image studies^22^ (Table 1).

Overall, both the observational studies and experimental studies provide evidence that adherence does matter, although the effect is weaker for RCT comparisons between LAIs and oral medications, probably because adherence is good for both interventions in these studies.

### Does Duration Matter?

**Figure 3:**
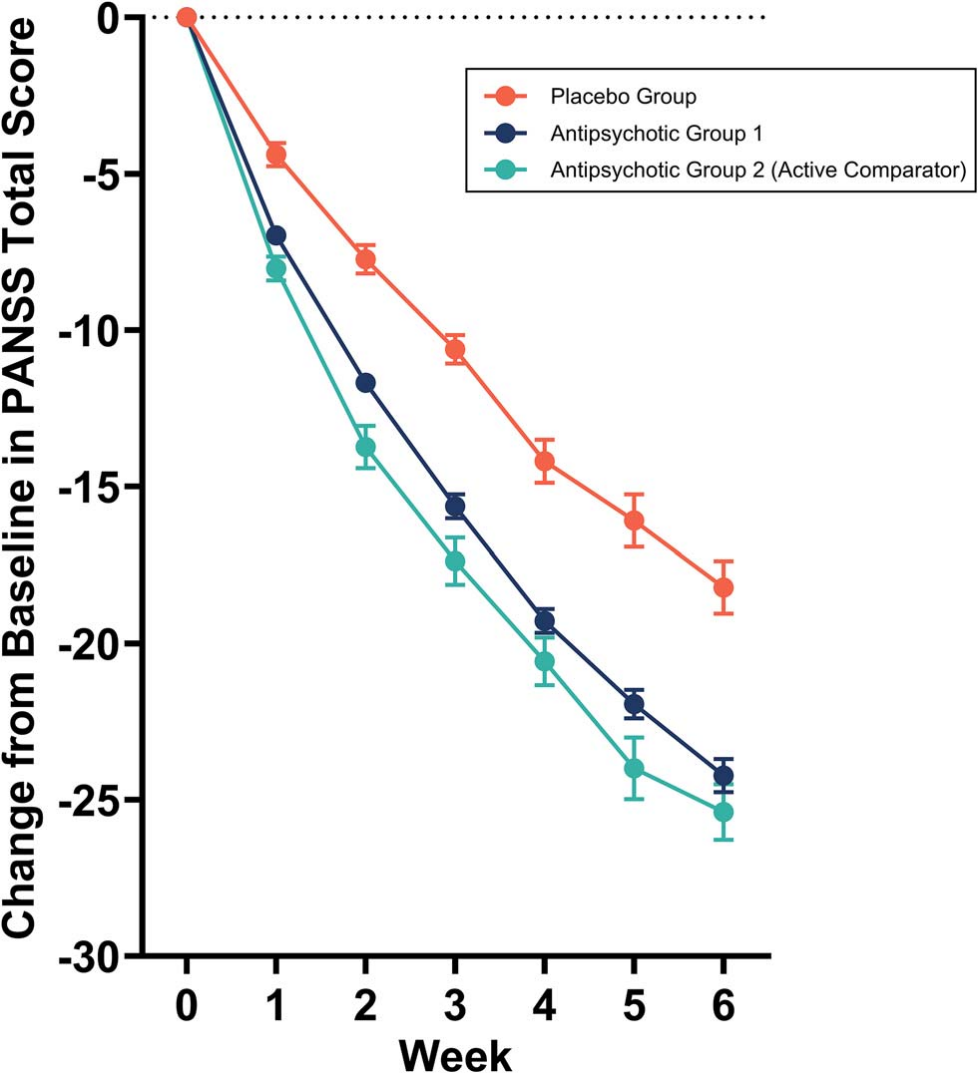
Global change from baseline in positive and negative syndrome scale (PANSS) score across 32 clinical trials of eight atypical antipsychotics.^51^ Values are mean ± SEM. Data were extracted from Younis et al.^51^ using WebPlot digitizer (version 5.2, Automeris LLC; California, United States) and plotted in GraphPad Prism (version 10.2.3, GraphPad software; Massachusetts, United States).

The relationship between duration of treatment and response was assessed in an analysis of 32 acute-phase, double-blind, placebo-controlled RCTs in schizophrenia (*n* = 14 219).^51^ This shows that although statistically significant separation from placebo is detectable after a week of antipsychotic treatment, the proportion of patients receiving antipsychotic treatment achieving at least a minimally clinically significant response (defined as >20% reduction in PANSS total from baseline) increases from approximately 15% at week 1 to approximately 55% at week 6, and that the mean reduction in total PANSS increases by a similar factor from approximately seven points after 1 week to approximately 25 points after 6 weeks (Figure 3).^51^ Although this analysis included both fixed and flexible-dose studies, which could confound duration effects, a prior analysis found that the time course of symptom response was similar in fixed and flexible-dose studies.^52^ The duration/symptom improvement relationship had a hyperbolic shape over the 6-week duration of the included trials (Figure 3).

In keeping with prior meta-analyses, the greatest reduction in symptoms was seen in the first 2 weeks of treatment.^51-53^ Prior meta-analyses of double-blind trials of longer duration in acute exacerbations of schizophrenia show that durations of greater than 6 weeks are not associated with statistically significant increases in efficacy in comparison to 6-week trials, and that the time-response curve begins to flatten after 4 weeks, with approximately 70% of the reduction in total symptoms seen at 1 year achieved after 4 weeks.^1^^,^^52^

In people with stable, chronic schizophrenia, meta-analysis of double-blind RCTs (k = 11, *n* = 2826) shows that antipsychotic withdrawal is associated with a 50% worsening in total symptoms at 1-year follow-up, in comparison to 10% for antipsychotic continuation.^54^ There was little change in total symptom severity over time in patients randomized to continue antipsychotic treatment, whereas patients randomized to antipsychotic withdrawal experienced gradually worsening symptoms over time, although the curve began to flatten after approximately 9 months.^54^ The significant benefit of antipsychotic maintenance in comparison to antipsychotic withdrawal for total symptom severity was first evident at 12 weeks.^54^ Meta-analysis of similarly designed studies in people with remitted FEP demonstrates that the benefit of maintenance antipsychotic treatment in comparison to antipsychotic withdrawal on relapse rates is first seen at 8 weeks.^17^

The evidence from RCTs reviewed above shows that duration of antipsychotic treatment is relevant to outcome. On average, the greatest reduction in symptoms in acute psychosis is seen in the first 2 weeks following initiation, and there is also evidence that symptomatic improvements begin to plateau approximately 4 weeks after initiation. The association of continuous antipsychotic treatment with less severe symptoms, reduced rates of relapse, hospitalization, and death in long-term studies, as discussed above, is further evidence of benefits of a longer duration of treatment.

### Summary and Implications

The main findings from meta-analyses included in this review on the relationships between these measures of inadequate treatment and outcomes are summarized in Table 1. As summarized in Table 1 and reviewed above, this evidence demonstrates that inadequate antipsychotic treatment (whether due to low dose and/or inadequate duration and/or poor adherence and/or no antipsychotic) throughout the course of psychotic illness is associated with a range of poor outcomes, including increased risk of relapse, more severe overall and positive symptoms, poorer functioning, increased use of inpatient psychiatric services, poorer quality-of-life and higher mortality. Many of these relationships were based on meta-analyses that either exclusively or predominantly included double-blind RCTs, suggesting that these relationships are likely to be causal, and were largely consistent with findings from observational studies, indicating that the results are also generalisable to other settings. These, therefore, represent important, potentially modifiable influences on outcome in psychosis.

A number of studies have investigated how common some of these factors are in clinical settings. Meta-analysis of observational studies conducted around the world estimates the rates of medication non-adherence to be 56% in schizophrenia (k = 9, *n* = 2643), and 44% in bipolar disorder (k = 10, *n* = 73 250).^55^ Observational data suggest that rates of prescription of antipsychotics in schizophrenia at sub-therapeutic doses are between 35% and 48% (depending on the setting and whether the threshold was set at ~250 or 300 mg chlorpromazine-equivalent dose/day), and approximately 60% in bipolar disorder.^56^^,^^57^ Thus, inadequate treatment is likely to be common in routine practice. Taken with our findings showing inadequate treatment is related to poor outcomes, this indicates that a key aim of clinical services should be to address inadequate treatment by evaluating its prevalence and intervening to reduce it.

However, of the studies included in this review, none considered the prevalence or impact on outcomes of all factors leading to inadequate treatment, and almost all considered only one factor at a time. This is a limitation as the factors are conceptually linked: poor adherence, sub-therapeutic dose, short duration of treatment, or periods of no treatment all mean insufficient treatment. As such, they could be considered together to determine the prevalence of inadequate treatment and whether this relates to outcomes. The potential advantage of this is that it provides a single metric. The concept of minimally adequate treatment has been used by the World Health Organisation (WHO) to evaluate services and treatment for anxiety, mood, and substance misuse disorders globally for approximately 25 years.^10^^,^^58^ There are no comparable investigations or criteria for what constitutes minimally adequate treatment for psychotic disorders. In addition to the evaluation of services, measuring inadequate treatment in psychosis would also help identify socio-demographic, service design, or illness variables associated with inadequate treatment, and support the development of interventions to address it. For example, there is already some evidence that one marker of inadequate treatment (adherence) can be improved in some settings, for example, with automated reminders or psychoeducation.^59^

### Defining Minimally Adequate Treatment of Psychosis

A barrier to efforts to quantify and reduce inadequate treatment is a lack of criteria for what constitutes minimally adequate treatment. This is a key issue is as research on prevalence, associations with outcomes, and strategies for improvement will be more reproducible if consistent criteria are used, as has been implemented for treatment resistance and remission, and has been raised for DUP.^6^^,^^11^^,^^60^ As discussed above, poorer outcomes are seen in psychosis when;

1) No antipsychotic is prescribed
2) Adherence is poor
3) Antipsychotic treatment is given at an inadequate dose
4) Antipsychotic treatment is given for an inadequate duration

#### Minimally Adequate Acute Treatment

Based on the studies included in this review, the minimally adequate treatment required to achieve response in an acute psychotic episode could be considered to be 2 mg risperidone-equivalent per day (250 mg chlorpromazine-equivalent per day) given for at least 1 week, with adherence of 75% or greater. This is based on meta-analysis of double-blind RCTs showing that this is the lowest dose and duration at which separation from placebo is detectable in acute exacerbations of schizophrenia spectrum disorders, and an observational study showing that partial adherence below 75% of prescribed doses is associated with poorer outcomes.^36^^,^^41^^,^^51^

However, the utility of this measure as a target for services is doubtful, as although separation from placebo is evident after a week, only 15% of people achieve at least a minimally clinically meaningful response at this time point.^30^ It is likely to be more useful to target a dose and duration associated with response in the majority of cases. In addition to this, all studies included in the dose–response meta-analysis had a duration of at least 4 weeks, so this is the shortest duration for which there is meta-analytic evidence to support the efficacy of the minimum effective dose. Taking this into account, at the group level, minimally adequate treatment required for response in acute exacerbations of psychosis can be defined as 2 mg risperidone-equivalent per day (250 mg chlorpromazine-equivalent per day) given for at least 4 weeks, with 75% adherence or greater. Meta-analytic evidence shows that this represents the dose at which 50% of the maximal response is achieved, and the duration of treatment following which more than 50% of people achieve at least a minimally clinically significant response.^36^^,^^51^

#### Minimally Adequate Maintenance Treatment

In patients already stabilized on antipsychotic treatment, minimally adequate treatment can be defined as 2 mg risperidone-equivalent per day (250 mg chlorpromazine-equivalent per day), with a maximum continuous treatment interruption or period of <75% adherence of 4 weeks. This is based on meta-analytic evidence in this population showing that 2 mg risperidone equivalent daily is the dose associated with approximately 50% of the maximal reduction in relapse rates, and that 4 weeks is the maximum treatment interruption not associated with an increased risk of relapse on average.^17^^,^^38^

#### Comparison with Existing Criteria

The Treatment Response and Resistance in Psychosis (TRRIP) consensus guidelines describe operationalized criteria for establishing treatment resistance in schizophrenia. They classify periods with less than 600 mg/day chlorpromazine-equivalent per day (~5 mg risperidone-equivalent per day) at less than 80% adherence as non-adherent, and trials of less than 6 weeks duration as inadequate trials.^11^ The criteria proposed here for minimally adequate treatment differ in several important ways. Whilst the near maximally effective dose is appropriate for a determination as to whether a treatment has failed, the minimally effective dose/ED50 is conceptually more appropriate for determining whether a treatment is minimally adequate. Similarly, whilst a trial of 6 weeks is appropriate to determine whether a treatment has failed, it is excessive to say whether it is minimally adequate, as more than 50% of people achieve at least a minimally clinically significant response after 4 weeks.^51^ The TRRIP guidelines also provide an approach to measure and categorize adherence (based on at least 2 sources, eg, pill counts, patient/carer reports, dispensing records, antipsychotic plasma levels), although the 80% threshold for adherence was based only on expert opinion. Here, we recommend an evidence based 75% threshold for adherence.^41^ Nevertheless, we still recommend determining adherence according to the prior consensus in the TRRIP guidelines, as this measure is already familiar, and measurement of adherence has not been widely adopted.^11^^,^^42^

### Does Duration of Inadequate Treatment (DIT) Matter?

In addition to the above evidence that inadequate treatment as a binary construct is linked to poorer outcomes, the studies showing an association between DUP and poorer outcomes raise the question of whether a longer DIT also matters.^6^ We were unable to identify any studies that assessed this across all measures of inadequate treatment, although a few studies found that a longer duration of single measures of inadequate treatment (such as partial-adherence or inadequate dose) was associated with greater risk of relapse.^17^^,^^32^^,^^33^ This means that it is unclear whether a longer DIT is related to other important outcomes such as symptom severity, quality of life or functioning. Such a relationship seems likely, based on the relationship between a longer DUP and these outcomes, and associations between single measures of inadequate treatment and these outcomes in analyses that considered single measures of inadequate treatment to be a time-invariant predictor,^6^ but it needs testing. As is the case in DUP research, confounders such as baseline illness severity may moderate this relationship.^6^

The lack of studies on the impact of the DIT is surprising, given the wealth of studies investigating DUP, but may be partly due to the influential idea that there is a “critical window” to treat psychotic disorders as soon as possible after their onset.^61^ However, the data reviewed above in patients with chronic illness indicates that inadequate treatment is still an important determinant of outcomes even years after the first onset of the illness.

The evidence for the association between long DUP and poor outcomes contributed to services in many healthcare settings focusing on reducing DUP^8^. Some healthcare systems have even set service targets for DUP, and evaluate services on the basis of the length of DUP^64^. For example, national guidelines in England recommend that adults experiencing a FEP should start treatment within 2 weeks of referral, and the WHO recommends to target a mean DUP of less than 3 months.^62^^,^^63^ This highlights the potential service implications of understanding if DIT is related to outcomes. Moreover, the association with service use suggests that reducing the DIT may be cost-effective. In addition to this, testing the association between DIT and outcomes in recently remitted FEP may help to clarify questions around optimal treatment strategies in this population.

### Operationalizing Duration of Inadequate Treatment (DIT)

As discussed above, antipsychotic treatment using an insufficient dose (<2 mg risperidone-equivalent per day/250 mg chlorpromazine-equivalent per day), duration (<4 weeks for response in acute psychosis, continuous treatment break >4 weeks for relapse prevention in stable patients), or with insufficient adherence (<75% adherence) is likely to be inadequate. Moreover, a longer DIT is likely to be associated with poorer outcomes, but this has not been tested. To address this, potential operationalized criteria for the DIT are discussed below:

1) DIT_(basic)_. It may not be feasible to reliably assess adherence in all clinical settings or in existing datasets, as patient/clinician reports notoriously overestimate adherence, and alternatives, such as technologies to monitor adherence, are not widely used in clinical practice.^42^^,^^64^ The most pragmatic definition of DIT therefore omits clinical assessment of medication taking, and consists of:
*Duration without antipsychotic treatment: sum of days with no antipsychotic prescribed or overt non-adherence (*eg*, not collected, documented refusal in inpatient setting).*
*Duration of inadequate acute antipsychotic treatment: sum of days following antipsychotic (re)initiation where dose does not reach ≥ ED50 (2 mg risperidone-equivalent/250 mg chlorpromazine-equivalent per day)* ^35^  *for a period of at least four continuous weeks.*
*Duration of inadequate maintenance antipsychotic treatment: Sum of on-treatment days following acute trial where antipsychotic dose is <ED50. Maintenance treatment is considered to have ended after an interruption of >4 continuous weeks.*

This measure is objective and can be calculated relatively easily from routinely collected pharmacy data.

2) DIT_(adherence)_. DIT (basic) is extended to incorporate adherence. During acute treatment, any 4-week period with <75% adherence is counted toward DIT. During maintenance treatment, adherence <75% for >4 consecutive weeks is considered to terminate maintenance treatment. Days of non-adherence are not counted in (b) or (c) if already counted in (a).

We propose measuring the DIT from when antipsychotic treatment is first initiated, as this is generally considered to represent the end of the DUP, although this could be adapted to commence from an alternative DUP endpoint for use in existing datasets in the minority of studies which require a brief period of antipsychotic treatment (eg, 7-28 days) before considering DUP to have ended.^6^ These measures and their recommended applications are summarized in Table 2.

**Table 2:** Recommended DIT Definitions in Different Settings (Calculated from First Initiation of Antipsychotic Treatment Onwards)

**Measure**	**Description**	**Advantages**	**Disadvantages**	**Recommended use**
**DIT** _**(basic)**_	Pragmatic definition of DIT, which omits clinical assessment of medication taking. Sum of: *A: Duration without antipsychotic treatment* *B: Duration of inadequate acute antipsychotic treatment* *C: Duration of inadequate maintenance antipsychotic treatment*	Easy to calculate	Does not consider covert non-adherence	Routine clinical practice
**DIT** _**(adherence)**_	DIT (basic) extended to incorporate adherence. Sum of: *A2: Duration without antipsychotic treatment* *B2: Duration of inadequate acute antipsychotic treatment* *C2: Duration of inadequate maintenance antipsychotic treatment*	Modification of existing TRRIP criteria^11^ Includes all measures of inadequate treatment	Challenge in assessing adherence in routine clinical practice	Services with capacity for measuring adherence Research settings

A: sum of days with no antipsychotic prescribed or overt non-adherence (eg, not collected, documented refusal in inpatient setting); B: sum of days following antipsychotic (re)initiation where dose does not reach ≥ED50 (2 mg risperidone-equivalent/250 mg chlorpromazine-equivalent per day)^35^ for a period of at least 4 continuous weeks; C: Sum of on-treatment days following acute trial where antipsychotic dose is <ED50. Maintenance treatment is considered to have ended after an interruption of >4 continuous weeks

A2: sum of days with no antipsychotic prescribed or overt non-adherence (eg, not collected, documented refusal in inpatient setting). Days of non-adherence are not counted in B2 or C2 if already counted in A2; B2: sum of days following antipsychotic (re)initiation where dose does not reach ≥ED50 (2 mg risperidone-equivalent/250 mg chlorpromazine-equivalent per day)^35^ and adherence does not reach ≥75% for a period of at least 4 continuous weeks; C2: Sum of on-treatment days following acute trial where antipsychotic dose is <ED50. Maintenance treatment is considered to have ended after an interruption of >4 continuous weeks or a period with <75% adherence for >4 consecutive weeks.

Abbreviation: DIT = duration of inadequate treatment.

### Limitations and Uncertainties

There are several relevant limitations of the evidence used to define minimally adequate treatment/DIT. We aimed to provide a clinician-focused narrative overview of the latest meta-analytic evidence and to propose criteria for the adequate treatment of psychosis. This was not a systematic review with an exhaustive search strategy, and so it is possible that relevant meta-analyses were missed. Nevertheless, the consistency and strength of the relationships demonstrated across several outcomes suggest that this is unlikely to change our overall conclusions. Additionally, our searches of the reference lists of identified articles partially mitigate against this, and our approach is also consistent with prior guidelines for the treatment of schizophrenia.^65^

We recognize that our putative criteria are based predominantly on the prevention/treatment of psychotic symptoms, as this is what the majority of studies focused on. It is possible that other outcomes differ in their association with inadequate treatment. For example, in contrast to relapse, which was associated with all markers of inadequate treatment, we found that outcomes such as mortality and quality-of-life were not consistently associated with each individual marker of inadequate treatment. Alternatively, this may be explained by small sample sizes for these comparisons (as the majority of research focused on relapse) and issues in interpreting LAI vs oral RCTs, as discussed above. Nevertheless, we did not find any association between negative symptom severity and any measure of inadequate antipsychotic treatment. This suggests that adequate treatment may not be useful in predicting negative symptom response, in keeping with the current consensus that there are no available effective treatments for negative symptoms.^5^^,^^7^^,^^65^ In addition, although 4 weeks is the maximum treatment interruption not associated with an increased risk of relapse in patients stabilized on antipsychotic treatment, it is not clear if the risk of relapse (or other poor outcomes) is elevated in the longer term if treatment is resumed within this time.^38^ We also acknowledge that the threshold for the degree of adherence which is adequate is based on a single prospective study, as we could not find any relevant meta-analysis, and that the analysis of the duration/symptom improvement relationship was based on analysis of a clinical trial database, with no report of a systematic search/study selection strategy, meaning that relevant data may have been excluded from the quantitative synthesis.

It is also important to consider whether antipsychotic treatment alone constitutes minimally adequate treatment. We acknowledge debate on this point, but highlight several recent umbrella reviews and meta-analyses which conclude that other treatments, including psychosocial interventions and brain stimulation, require more robust evidence on their long-term, clinically meaningful benefits on outcomes in psychosis.^66-69^ As such, we anticipate that the criteria we propose may need updating to include other treatments with the accrual of new evidence.

A further consideration is that, unlike DUP, which draws a binary distinction between treated and untreated psychosis, DIT involves both a duration and a degree component to inadequate treatment. This is a potential limitation of the DUP concept as it is generally applied, as it assumes any antipsychotic is sufficient. Related to this, it should be acknowledged that there are several approaches to determining dose equivalencies. Research studies could investigate the importance of these issues.

The precise mechanism by which inadequate treatment may result in poorer outcomes is unclear.^70^^,^^71^ In the DUP literature, it has been hypothesized that active psychosis leads to poorer outcomes due to direct neurotoxic effects, and/or that it is socially toxic (i.e., that the stigma associated with psychotic symptoms leads to longstanding disruption of relationships, opportunities, etc.).^72^^,^^73^ Using our DIT criteria, future studies could further test these hypotheses beyond the first episode in people with chronic psychotic illnesses. It is also possible that psychotic symptoms are not necessary to explain the hypothesized relationship between a longer DIT and poorer outcomes. For example, antipsychotic treatment has been theorized to be neuroprotective, potentially due to anti-inflammatory effects or modulation of brain volumes.^27^^,^^74^^,^^75^ Inclusion of a symptom threshold with DIT, as discussed below, would allow investigation of whether active symptoms mediate the hypothesized relationship between a longer DIT and poorer outcomes.

#### Extrapolating Group Average Responses to Individuals

A key consideration is that the criteria proposed are based on average responses, and have been developed largely from people diagnosed with schizophrenia spectrum disorders. This neglects that individual patient factors, including pharmacokinetics, may affect treatment response.^76^ For example, meta-analysis of individual patient data from single and double-blind RCTs shows that overall symptom reduction is greater in women with schizophrenia compared to men, at similar average antipsychotic doses.^77^ One potential interpretation of these data is that the minimum effective dose may be lower in women, but this has not been tested and is a general limitation of the current meta-analytic evidence available to guide treatment decisions in schizophrenia.^36^^,^^38^

A related point is whether the minimally adequate treatment of other psychotic disorders, such as bipolar disorder, differs from that of schizophrenia. A recent meta-analysis of double-blind RCTs tested this by comparing findings from antipsychotic trials conducted in people with FEP (which includes people with psychosis related to bipolar and other non-schizophrenia disorders) to trials conducted in people with schizophrenia.^78^ This showed that the effectiveness and side-effect profile of antipsychotic treatment in people with FEP (and also in children and adolescents, older people, people with co-morbid substance abuse, and people with treatment-resistant schizophrenia) is similar to that seen in people of working age experiencing acute exacerbations of chronic schizophrenia.^78^ This provides justification for extrapolating criteria based on schizophrenia trials to FEP and these other subgroups. We also chose to pragmatically apply the above criteria to other psychotic disorders, as we could not identify meta-analytic evidence on the minimum effective antipsychotic dose or duration for other psychotic disorders, and our review shows that measures of inadequate treatment are associated with poorer outcomes across psychotic disorders.^17^^,^^20^^,^^32^^,^^33^ Moreover, common criteria are consistent with the existing literature on DUP, which uses the same definition across psychotic disorders.^6^ As there is some evidence that diagnosis moderates the relationship between DUP and outcomes, it would be useful for studies assessing the relationship between adequate treatment/DIT and outcomes to report results separately by diagnosis in addition to pooled results.^6^

Another related conceptual issue with the above measures is that the framing of inadequate treatment as a predictor of poor outcome without reference to current presentation implies that all people with psychotic disorders benefit from continuous, lifelong antipsychotic treatment. However, as discussed above, some people with psychotic disorders exhibit good outcomes at long follow-up with subtherapeutic or no antipsychotic treatment.^33^^,^^34^ Inclusion of these people would weaken any group-level associations between DIT and poor outcomes. As precision medicine approaches have not been widely implemented in medicine or psychiatry, extrapolating from average responses to the individual is a limitation of evidence-based psychiatry in general at present.^79^ Inclusion of a symptom threshold may help to address this.

A more important consideration is that our criteria may be seen to advocate treatment, with a risk of adverse effects, when it is potentially unnecessary.^26^ Some of these (such as Type 2 Diabetes Mellitus, metabolic syndrome, and tardive dyskinesia) are potentially irreversible.^26^^,^^80^^,^^81^ Further studies to identify predictors of good outcomes following antipsychotic discontinuation would help to address these issues.^40^^,^^82^ However, on the other hand, one observational study found that almost half of patients referred to a tertiary service for management of what was considered to be treatment-resistant psychosis had either undetectable (19%) or subtherapeutic (25%) antipsychotic plasma levels.^83^ Although this real-world data supports our suggestion that a greater focus on reducing inadequate antipsychotic treatment could lead to improved outcomes, we do not intend to advocate a fixed minimum dose/duration for all patients in real-life clinical practice. As discussed above, there may be good grounds to prescribe lower doses/durations in individual cases, although we highlight that the existing literature shows that this is likely be associated with poorer outcomes on average. It should therefore have a clear justification, for example, the presence of genetic polymorphisms known to be associated with different dose requirements.^76^ Similarly, as our thresholds were based on antipsychotic doses/durations where approximately 50% of patients will experience a clinically meaningful response, it is also important to note that approximately 50% will not do so. Our thresholds set criteria for minimally adequate treatment at the group level but are not criteria for optimized treatment at the group or individual level, and may be sub-optimal for many patients. Therefore, they should not be interpreted as a recommendation not to increase antipsychotic dose/duration where there are on-going symptoms. Clinical judgment and complementary guidelines on treatment resistance should be followed in these circumstances.^11^

#### Comparison with the Duration of Active Psychosis after Treatment Is Initiated (DAT)

The duration of active psychotic symptoms after treatment is initiated (DAT) is calculated by summing all periods with psychotic symptoms over a threshold following the FEP. This overlaps conceptually to some extent with our proposal, but it has important limitations. A recent scoping review found that there have been a total of eight original studies (on three overlapping samples) on the relationship between DAT and outcomes, published between 2015 and 2024.^84^ There were 1-2 studies per outcome across a wide range of outcomes, including negative symptoms, neurocognition, and pro-inflammatory cytokine levels.^84^ Although the studies originated from only three different research groups, there was nevertheless a lack of consistency in the assessment measures and symptom thresholds used to calculate the DAT.^84^ This limitation is unsurprising, as there are no operationalized criteria for the DAT.

A more important conceptual issue is that none of the studies included in the scoping review included any measure of treatment at the time of active symptoms.^84^ The DAT, as applied, is therefore a longitudinal measure of psychotic illness severity (using a binary threshold to determine whether symptoms are active or not); considering this, it is more likely to be confounded with baseline illness severity than our proposed measure. Furthermore, the DAT has limited utility if treatment is adequate (although this is not measured by it), as in this circumstance, it overlaps with the definition of treatment resistance; in fact, once criteria for treatment resistance are met, the DAT is likely to be very close to the delay to clozapine. Our alternative proposal to only consider supra-threshold symptoms when treatment is inadequate has the considerable benefit of demonstrating not only that there are clinically meaningful psychotic symptoms for that individual, but that this is related to the modifiable factor of inadequate treatment.

For these reasons, although our proposed DIT criteria are not mutually exclusive with measuring active symptoms, we propose doing so only when combined with a measure of treatment adequacy. Any of the prior definitions of DIT could be combined with a symptom threshold criterion (eg, consensus remission criteria) in this way, although this would require regular assessment of symptoms, and it is circular for outcomes related to psychotic symptoms severity, as both the predictor and outcome include this. We recommend this wherever possible, but do not recommend a specific threshold here as our main aim was to propose evidence-based criteria which could be easily adopted in a range of settings, including services which may not have the resources to regularly assess symptoms. This approach is consistent with the WHO approach for evaluating the treatment of depression and other common psychiatric disorders,^10^^,^^58^ and is likely to have considerable utility at the group/service level, as our criteria are based on the average responses of tens of thousands of people with psychosis.

A further advantage of our proposal in comparison to the DAT is that studies on DUP generally do not include a symptom threshold.^6^ Our main criteria for DIT are therefore more conceptually aligned with DUP research; this is critical, as our aim was to propose criteria that test whether the principle underlying the relationship between DUP and outcomes (that delays in treatment initiation are related to poorer outcomes in the first episode) is also applicable throughout the entire illness course.

## Conclusion and Future Directions

In this narrative review, we synthesized and appraised evidence linking inadequate antipsychotic treatment to outcomes across the course of psychotic illness. We show that inadequate antipsychotic treatment (whether due to inadequate dose/duration, non-compliance, or non-prescription of antipsychotic) was repeatedly associated with poorer outcomes across a range of patient-centered outcome measures. Many of these relationships, including functional outcomes, relapse, quality-of-life, total symptom and positive symptom severity, were based on meta-analyses which either exclusively or predominantly included double-blind RCTs, suggesting that these relationships are likely to be causal.

We further demonstrated the relevance of this by showing that inadequate treatment is common, and highlighted that, despite their high prevalence and importance for clinical outcomes, services do not routinely measure inadequate treatment, and are not evaluated on this basis. We therefore conclude that inadequate treatment is a neglected, but modifiable, prognostic factor in psychosis. To address this, we suggest routine measurement of the adequacy of treatment, and we propose evidence-based criteria that capture all factors contributing to adequate treatment under a single outcome measure. We go on to suggest that a longer DIT could be an important factor for clinical outcomes, based on previous studies associating a longer DUP with poorer outcomes, and set out putative operationalized criteria for DIT for use in different settings.

In conclusion, evidence indicates that low dose, poor adherence, and short duration of antipsychotic treatment are modifiable factors associated with poorer outcomes, and are prevalent in clinical settings. We identify thresholds for these factors that can be used to define inadequate treatment. The impact of the DIT on outcomes remains unclear and warrants research. We propose operationalized criteria to more reliably assess the scale and extent of the problem, evaluate services, assess relationships with outcomes, and support the development of interventions to reduce inadequate treatment.

## References

[1] Leucht S, Leucht C, Huhn M, et al. Sixty years of placebo-controlled antipsychotic drug trials in acute schizophrenia: systematic review, Bayesian meta-analysis, and meta-regression of efficacy predictors. *Am J Psychiatry*. 2017;174:927-942. 10.1176/appi.ajp.2017.16121358 28541090

[2] Schneider-Thoma J, Chalkou K, Dorries C, et al. Comparative efficacy and tolerability of 32 oral and long-acting injectable antipsychotics for the maintenance treatment of adults with schizophrenia: a systematic review and network meta-analysis. *Lancet.* 2022;399:824-836. 10.1016/S0140-6736(21)01997-8 35219395

[3] Huhn M, Nikolakopoulou A, Schneider-Thoma J, et al. Comparative efficacy and tolerability of 32 oral antipsychotics for the acute treatment of adults with multi-episode schizophrenia: a systematic review and network meta-analysis. *Lancet.* 2019;394:939-951. 10.1016/S0140-6736(19)31135-3 31303314 PMC6891890

[4] Osugo M, Whitehurst T, Shatalina E, et al. Dopamine partial agonists and prodopaminergic drugs for schizophrenia: systematic review and meta-analysis of randomized controlled trials. *Neurosci Biobehav Rev*. 2022;135:104568. 10.1016/j.neubiorev.2022.104568 35131396

[5] Howes O, Fusar-Poli P, Osugo M. Treating negative symptoms of schizophrenia: current approaches and future perspectives. *Br J Psychiatry*. 2023;223:332-335. 10.1192/bjp.2023.57 37272623

[6] Howes OD, Whitehurst T, Shatalina E, et al. The clinical significance of duration of untreated psychosis: an umbrella review and random-effects meta-analysis. *World Psychiatry*. 2021;20:75-95. 10.1002/wps.20822 33432766 PMC7801839

[7] Osugo M, Whitehurst T, Erritzoe D, et al. Role of serotonin in the neurobiology of schizophrenia and association with negative symptoms. *JAMA Psychiatry*. 2026;83:185-195. 10.1001/jamapsychiatry.2025.3430 PMC12696662 41370075

[8] Salazar de Pablo G, Guinart D, Armendariz A, et al. Duration of untreated psychosis and outcomes in first-episode psychosis: systematic review and meta-analysis of early detection and intervention strategies. *Schizophr Bull*. 2024;50:771-783. 10.1093/schbul/sbae017 38491933 PMC11283197

[9] Jonas KG, Fochtmann LJ, Perlman G, et al. Lead-time bias confounds association between duration of untreated psychosis and illness course in schizophrenia. *Am J Psychiatry*. 2020;177:327-334. 10.1176/appi.ajp.2019.19030324 32046533 PMC10754034

[10] Santomauro DF, Vos T, Whiteford HA, Chisholm D, Saxena S, Ferrari AJ. Service coverage for major depressive disorder: estimated rates of minimally adequate treatment for 204 countries and territories in 2021. *Lancet Psychiatry*. 2024;11:1012-1021. 10.1016/S2215-0366(24)00317-1 39572105 PMC11579305

[11] Howes OD, McCutcheon R, Agid O, et al. Treatment-resistant schizophrenia: treatment response and resistance in psychosis (TRRIP) working group consensus guidelines on diagnosis and terminology. *Am J Psychiatry*. 2017;174:216-229. 10.1176/appi.ajp.2016.16050503 27919182 PMC6231547

[12] Bozzatello P, Bellino S, Rocca P. Predictive factors of treatment resistance in first episode of psychosis: a systematic review. *Front Psychiatry*. 2019;10:67. 10.3389/fpsyt.2019.00067 30863323 PMC6399388

[13] Jia N, Li Z, Li X, et al. Long-term effects of antipsychotics on mortality in patients with schizophrenia: a systematic review and meta-analysis. *Braz J Psychiatry*. 2022;44:664-673. 10.47626/1516-4446-2021-2306 36709510 PMC9851750

[14] Sturup AE, Nordentoft M, Jimenez-Solem E, et al. Discontinuation of antipsychotics in individuals with first-episode schizophrenia and its association to functional outcomes, hospitalization and death: a register-based nationwide follow-up study. *Psychol Med*. 2023;53:5033-5041. 10.1017/S0033291722002021 35818718

[15] Taipale H, Tanskanen A, Correll CU, Tiihonen J. Real-world effectiveness of antipsychotic doses for relapse prevention in patients with first-episode schizophrenia in Finland: a nationwide, register-based cohort study. *Lancet Psychiatry*. 2022;9:271-279. 10.1016/S2215-0366(22)00015-3 35182475

[16] Efthimiou O, Taipale H, Radua J, et al. Efficacy and effectiveness of antipsychotics in schizophrenia: network meta-analyses combining evidence from randomised controlled trials and real-world data. *Lancet Psychiatry*. 2024;11:102-111. 10.1016/S2215-0366(23)00366-8 38215784

[17] Kishi T, Ikuta T, Matsui Y, et al. Effect of discontinuation v. maintenance of antipsychotic medication on relapse rates in patients with remitted/stable first-episode psychosis: a meta-analysis. *Psychol Med*. 2019;49:772-779. 10.1017/S0033291718001393 29909790

[18] Ostuzzi G, Vita G, Bertolini F, et al. Continuing, reducing, switching, or stopping antipsychotics in individuals with schizophrenia-spectrum disorders who are clinically stable: a systematic review and network meta-analysis. *Lancet Psychiatry*. 2022;9:614-624. 10.1016/S2215-0366(22)00158-4 35753323

[19] Hojlund M, Kemp AF, Haddad PM, Neill JC, Correll CU. Standard versus reduced dose of antipsychotics for relapse prevention in multi-episode schizophrenia: a systematic review and meta-analysis of randomised controlled trials. *Lancet Psychiatry*. 2021;8:471-486. 10.1016/S2215-0366(21)00078-X 34023019

[20] Alvarez-Jimenez M, Priede A, Hetrick SE, et al. Risk factors for relapse following treatment for first episode psychosis: a systematic review and meta-analysis of longitudinal studies. *Schizophr Res*. 2012;139:116-128. 10.1016/j.schres.2012.05.007 22658527

[21] De Hert M, Sermon J, Geerts P, Vansteelandt K, Peuskens J, Detraux J. The use of continuous treatment versus placebo or intermittent treatment strategies in stabilized patients with schizophrenia: a systematic review and meta-analysis of randomized controlled trials with first- and second-generation antipsychotics. *CNS Drugs*. 2015;29:637-658. 10.1007/s40263-015-0269-4 26293744

[22] Kishimoto T, Hagi K, Kurokawa S, Kane JM, Correll CU. Long-acting injectable versus oral antipsychotics for the maintenance treatment of schizophrenia: a systematic review and comparative meta-analysis of randomised, cohort, and pre–post studies. *Lancet Psychiatry*. 2021;8:387-404. 10.1016/S2215-0366(21)00039-0 33862018

[23] Takeuchi H, MacKenzie NE, Samaroo D, Agid O, Remington G, Leucht S. Antipsychotic dose in acute schizophrenia: a meta-analysis. *Schizophr Bull*. 2020;46:1439-1458. 10.1093/schbul/sbaa063 32415847 PMC7707077

[24] Olagunju AT, Clark SR, Baune BT. Long-acting atypical antipsychotics in schizophrenia: a systematic review and meta-analyses of effects on functional outcome. *Aust N Z J Psychiatry*. 2019;53:509-527. 10.1177/0004867419837358 30957510

[25] Osugo M, Wall MB, Selvaggi P, et al. Striatal dopamine D2/D3 receptor regulation of human reward processing and behaviour. *Nat Commun*. 2025;16:1852. 10.1038/s41467-025-56663-7 39984436 PMC11845780

[26] Pillinger T, McCutcheon RA, Vano L, et al. Comparative effects of 18 antipsychotics on metabolic function in patients with schizophrenia, predictors of metabolic dysregulation, and association with psychopathology: a systematic review and network meta-analysis. *Lancet Psychiatry*. 2020;7:64-77. 10.1016/S2215-0366(19)30416-X 31860457 PMC7029416

[27] Selvaggi P, Osugo M, Zahid U, et al. Antipsychotics cause reversible structural brain changes within one week. *Neuropsychopharmacology.* 2025;50:1275-1283. 10.1038/s41386-025-02120-4 40335667 PMC12170900

[28] Chapman GE, Osugo M, de Marvao A, Howes OD. Aripiprazole-associated QT prolongation in a healthy study volunteer: a case report and literature review. *J Clin Psychopharmacol*. 2024;44:591-594. 10.1097/JCP.0000000000001921 39442546

[29] Osugo M, Zahid U, Selvaggi P, et al. Effects of antipsychotics on human cognitive function: causal evidence from healthy volunteers following sustained D2/D3 antagonism, D2/D3 partial agonism and placebo. *Mol Psychiatry*. 2025;30:5315-5325. 10.1038/s41380-025-03116-8 40684007 PMC12532602

[30] Vita A, De Peri L, Deste G, Barlati S, Sacchetti E. The effect of antipsychotic treatment on cortical gray matter changes in schizophrenia: does the class matter? A meta-analysis and meta-regression of longitudinal magnetic resonance imaging studies. *Biol Psychiatry*. 2015;78:403-412. 10.1016/j.biopsych.2015.02.008 25802081

[31] Fusar-Poli P, Smieskova R, Kempton MJ, Ho BC, Andreasen NC, Borgwardt S. Progressive brain changes in schizophrenia related to antipsychotic treatment? A meta-analysis of longitudinal MRI studies. *Neurosci Biobehav Rev*. 2013;37:1680-1691. 10.1016/j.neubiorev.2013.06.001 23769814 PMC3964856

[32] Thompson A, Winsper C, Marwaha S, et al. Maintenance antipsychotic treatment versus discontinuation strategies following remission from first episode psychosis: systematic review. *BJPsych Open*. 2018;4:215-225. 10.1192/bjo.2018.17 29988997 PMC6034451

[33] Moncrieff J, Crellin N, Stansfeld J, et al. Antipsychotic dose reduction and discontinuation versus maintenance treatment in people with schizophrenia and other recurrent psychotic disorders in England (the RADAR trial): an open, parallel-group, randomised controlled trial. *Lancet Psychiatry*. 2023;10:848-859. 10.1016/S2215-0366(23)00258-4 37778356

[34] Wunderink L, Nieboer RM, Wiersma D, Sytema S, Nienhuis FJ. Recovery in remitted first-episode psychosis at 7 years of follow-up of an early dose reduction/discontinuation or maintenance treatment strategy: long-term follow-up of a 2-year randomized clinical trial. *JAMA Psychiatry*. 2013;70:913-920. 10.1001/jamapsychiatry.2013.19 23824214

[35] Leucht S, Samara M, Heres S, Patel MX, Woods SW, Davis JM. Dose equivalents for second-generation antipsychotics: the minimum effective dose method. *Schizophr Bull*. 2014;40:314-326. 10.1093/schbul/sbu001 24493852 PMC3932104

[36] Leucht S, Crippa A, Siafis S, Patel MX, Orsini N, Davis JM. Dose-response meta-analysis of antipsychotic drugs for acute schizophrenia. *Am J Psychiatry*. 2020;177:342-353. 10.1176/appi.ajp.2019.19010034 31838873

[37] Sabe M, Zhao N, Crippa A, Kaiser S. Antipsychotics for negative and positive symptoms of schizophrenia: dose-response meta-analysis of randomized controlled acute phase trials. *NPJ Schizophr*. 2021;7:43. 10.1038/s41537-021-00171-2 34518532 PMC8438046

[38] Leucht S, Bauer S, Siafis S, et al. Examination of dosing of antipsychotic drugs for relapse prevention in patients with stable schizophrenia: a meta-analysis. *JAMA Psychiatry*. 2021;78:1238-1248. 10.1001/jamapsychiatry.2021.2130 34406325 PMC8374744

[39] Gardner DM, Murphy AL, O'Donnell H, Centorrino F, Baldessarini RJ. International consensus study of antipsychotic dosing. *Am J Psychiatry*. 2010;167:686-693. 10.1176/appi.ajp.2009.09060802 20360319

[40] Tani H, Takasu S, Uchida H, Suzuki T, Mimura M, Takeuchi H. Factors associated with successful antipsychotic dose reduction in schizophrenia: a systematic review of prospective clinical trials and meta-analysis of randomized controlled trials. *Neuropsychopharmacology.* 2020;45:887-901. 10.1038/s41386-019-0573-7 31770770 PMC7075912

[41] Subotnik KL, Nuechterlein KH, Ventura J, et al. Risperidone nonadherence and return of positive symptoms in the early course of schizophrenia. *Am J Psychiatry*. 2011;168:286-292. 10.1176/appi.ajp.2010.09010087 21205805 PMC5069345

[42] Kane JM, Kishimoto T, Correll CU. Non-adherence to medication in patients with psychotic disorders: epidemiology, contributing factors and management strategies. *World Psychiatry*. 2013;12:216-226. 10.1002/wps.20060 24096780 PMC3799245

[43] Yeisen RAH, Bjornestad J, Joa I, Johannessen JO, Opjordsmoen S. Experiences of antipsychotic use in patients with early psychosis: a two-year follow-up study. *BMC Psychiatry*. 2017;17:299. 10.1186/s12888-017-1425-9 28830453 PMC5567881

[44] Titus-Lay EN, Ansara ED, Isaacs AN, Ott CA. Evaluation of adherence and persistence with oral versus long-acting injectable antipsychotics in patients with early psychosis. *Ment Health Clin*. 2018;8:56-62. 10.9740/mhc.2018.03.056 29955546 PMC6007741

[45] Coldham EL, Addington J, Addington D. Medication adherence of individuals with a first episode of psychosis. *Acta Psychiatr Scand*. 2002;106:286-290. 10.1034/j.1600-0447.2002.02437.x 12225495

[46] Lin D, Thompson-Leduc P, Ghelerter I, et al. Real-world evidence of the clinical and economic impact of long-acting injectable versus Oral antipsychotics among patients with schizophrenia in the United States: a systematic review and meta-analysis. *CNS Drugs*. 2021;35:469-481. 10.1007/s40263-021-00815-y 33909272 PMC8144083

[47] Haddad PM, Kishimoto T, Correll CU, Kane JM. Ambiguous findings concerning potential advantages of depot antipsychotics: in search of clinical relevance. *Curr Opin Psychiatry*. 2015;28:216-221. 10.1097/YCO.0000000000000160 25785710

[48] Kane JM, Kishimoto T, Correll CU. Assessing the comparative effectiveness of long-acting injectable vs. oral antipsychotic medications in the prevention of relapse provides a case study in comparative effectiveness research in psychiatry. *J Clin Epidemiol*. 2013;66:S37-S41. 10.1016/j.jclinepi.2013.01.012 23849151 PMC3742035

[49] Kishimoto T, Robenzadeh A, Leucht C, et al. Long-acting injectable vs oral antipsychotics for relapse prevention in schizophrenia: a meta-analysis of randomized trials. *Schizophr Bull*. 2014;40:192-213. 10.1093/schbul/sbs150 23256986 PMC3885289

[50] Bossie CA, Alphs LD, Correll CU. Long-acting injectable versus daily oral antipsychotic treatment trials in schizophrenia: pragmatic versus explanatory study designs. *Int Clin Psychopharmacol*. 2015;30:272-281. 10.1097/YIC.0000000000000082 26049673 PMC4525810

[51] Younis IR, Gopalakrishnan M, Mathis M, et al. Association of end point definition and randomized clinical trial duration in clinical trials of schizophrenia medications. *JAMA Psychiatry*. 2020;77:1064-1071. 10.1001/jamapsychiatry.2020.1596 32609294 PMC7330825

[52] Leucht S, Busch R, Hamann J, Kissling W, Kane JM. Early-onset hypothesis of antipsychotic drug action: a hypothesis tested. *Confirmed and Extended Biological Psychiatry*. 2005;57:1543-1549. 10.1016/j.biopsych.2005.02.023 15953491

[53] Agid O, Seeman P, Kapur S. The "delayed onset" of antipsychotic action--an idea whose time has come and gone. *J Psychiatry Neurosci*. 2006;31:93-100. 16575424 PMC1413955

[54] Takeuchi H, Kantor N, Sanches M, Fervaha G, Agid O, Remington G. One-year symptom trajectories in patients with stable schizophrenia maintained on antipsychotics versus placebo: Meta-analysis. *Br J Psychiatry*. 2017;211:137-143. 10.1192/bjp.bp.116.186007 28522434

[55] Semahegn A, Torpey K, Manu A, Assefa N, Tesfaye G, Ankomah A. Psychotropic medication non-adherence and its associated factors among patients with major psychiatric disorders: a systematic review and meta-analysis. *Syst Rev*. 2020;9:17. 10.1186/s13643-020-1274-3 31948489 PMC6966860

[56] Sweileh WM, Odeh JB, Shraim NY, Zyoud SH, Sawalha AF, Al-Jabi SW. Evaluation of defined daily dose, percentage of British National Formulary maximum and chlorpromazine equivalents in antipsychotic drug utilization. *Saudi Pharm J*. 2014;22:127-132. 10.1016/j.jsps.2013.03.003 24648824 PMC3950502

[57] Hartung DM, Wisdom JP, Pollack DA, et al. Patterns of atypical antipsychotic subtherapeutic dosing among Oregon Medicaid patients. *J Clin Psychiatry*. 2008;69:1540-1547. 10.4088/jcp.v69n1003 19192436 PMC3155805

[58] Wang PS, Aguilar-Gaxiola S, Alonso J, et al. Use of mental health services for anxiety, mood, and substance disorders in 17 countries in the WHO world mental health surveys. *Lancet.* 2007;370:841-850. 10.1016/s0140-6736(07)61414-7 17826169 PMC2847360

[59] El Abdellati K, De Picker L, Morrens M. Antipsychotic treatment failure: a systematic review on risk factors and interventions for treatment adherence in psychosis. *Front Neurosci*. 2020;14:531763. 10.3389/fnins.2020.531763 33162877 PMC7584050

[60] Andreasen NC, Carpenter WT Jr, Kane JM, Lasser RA, Marder SR, Weinberger DR. Remission in schizophrenia: proposed criteria and rationale for consensus. *Am J Psychiatry*. 2005;162:441-449. 10.1176/appi.ajp.162.3.441 15741458

[61] Birchwood M, Todd P, Jackson C. Early intervention in psychosis. The critical period hypothesis. *Br J Psychiatry Suppl*. 1998;172:53-59. 9764127

[62] Psychosis and Schizophrenia in Adults . Quality Standard. National Institute for Health Care Excellence. https://www.nice.org.uk/guidance/qs80.

[63] Bertolote J, McGorry P. Early intervention and recovery for young people with early psychosis: consensus statement. *Br J Psychiatry Suppl*. 2005;48:s116-s119. 10.1192/bjp.187.48.s116 16055800

[64] Wu T, Xiao X, Yan S, et al. Digital health interventions to improve adherence to oral antipsychotics among patients with schizophrenia: a scoping review. *BMJ Open*. 2023;13:e071984. 10.1136/bmjopen-2023-071984 PMC10660841 37977861

[65] Barnes TR, Drake R, Paton C, et al. Evidence-based guidelines for the pharmacological treatment of schizophrenia: updated recommendations from the British Association for Psychopharmacology. *J Psychopharmacol*. 2020;34:3-78. 10.1177/0269881119889296 31829775

[66] Jauhar S, McKenna PJ, Radua J, Fung E, Salvador R, Laws KR. Cognitive-behavioural therapy for the symptoms of schizophrenia: systematic review and meta-analysis with examination of potential bias. *Br J Psychiatry*. 2014;204:20-29. 10.1192/bjp.bp.112.116285 24385461

[67] Berendsen S, Berendse S, van der Torren J, Vermeulen J, de Haan L. Cognitive behavioural therapy for the treatment of schizophrenia spectrum disorders: an umbrella review of meta-analyses of randomised controlled trials. *EClinicalMedicine.* 2024;67:102392. 10.1016/j.eclinm.2023.102392 38274116 PMC10809079

[68] Cella M, Roberts S, Pillny M, et al. Psychosocial and behavioural interventions for the negative symptoms of schizophrenia: a systematic review of efficacy meta-analyses. *Br J Psychiatry*. 2023;223:321-331. 10.1192/bjp.2023.21 36919340 PMC10331321

[69] Lorentzen R, Nguyen TD, McGirr A, Hieronymus F, Ostergaard SD. The efficacy of transcranial magnetic stimulation (TMS) for negative symptoms in schizophrenia: a systematic review and meta-analysis. *Schizophrenia (Heidelb)*. 2022;8:35. 10.1038/s41537-022-00248-6 35853882 PMC9261093

[70] Zahid U, McCutcheon RA, Borgan F, et al. The effect of antipsychotics on glutamate levels in the anterior cingulate cortex and clinical response: a (1)H-MRS study in first-episode psychosis patients. *Front Psychiatry*. 2022;13:967941. 10.3389/fpsyt.2022.967941 36032237 PMC9403834

[71] Zahid U, Osugo M, Selvaggi P, et al. The effects of dopamine receptor antagonist and partial agonist antipsychotics on the glutamatergic system: a double-blind, randomised, placebo-controlled 1H-MRS cross-over study in healthy volunteers. *Br J Psychiatry*. 2025;1-8. 10.1192/bjp.2025.10319 40698574

[72] Wyatt RJ . Neuroleptics and the natural course of schizophrenia. *Schizophr Bull*. 1991;17:325-351. 10.1093/schbul/17.2.325 1679255

[73] Norman RMG, Malla AK, Manchanda R. Is untreated psychosis socially toxic? *Early Intervention in Psychiatry*. 2007;1:267-270. 10.1111/j.1751-7893.2007.00038.x

[74] Chen AT, Nasrallah HA. Neuroprotective effects of the second generation antipsychotics. *Schizophr Res*. 2019;208:1-7. 10.1016/j.schres.2019.04.009 30982644

[75] Kim YK, Na KS. Neuroprotection in schizophrenia and its therapeutic implications. *Psychiatry Investig*. 2017;14:383-391. 10.4306/pi.2017.14.4.383 PMC5561394 28845163

[76] Hart XM, Grunder G, Ansermot N, et al. Optimisation of pharmacotherapy in psychiatry through therapeutic drug monitoring, molecular brain imaging and pharmacogenetic tests: focus on antipsychotics. *World J Biol Psychiatry*. 2024;25:451-536. 10.1080/15622975.2024.2366235 38913780

[77] Storosum BWC, Mattila T, Wohlfarth TD, et al. Gender differences in the response to antipsychotic medication in patients with schizophrenia: an individual patient data meta-analysis of placebo-controlled studies. *Psychiatry Res*. 2023;320:114997. 10.1016/j.psychres.2022.114997 36603382

[78] Leucht S, Chaimani A, Krause M, et al. The response of subgroups of patients with schizophrenia to different antipsychotic drugs: a systematic review and meta-analysis. *Lancet Psychiatry*. 2022;9:884-893. 10.1016/S2215-0366(22)00304-2 36228647

[79] Takamiya A, Kishimoto T. Is this the end of precision medicine? Or the beginning? *Lancet Psychiatry*. 2022;9:849-850. 10.1016/S2215-0366(22)00336-4 36228646

[80] Burschinski A, Schneider-Thoma J, Chiocchia V, et al. Metabolic side effects in persons with schizophrenia during mid- to long-term treatment with antipsychotics: a network meta-analysis of randomized controlled trials. *World Psychiatry*. 2023;22:116-128. 10.1002/wps.21036 36640396 PMC9840505

[81] Carbon M, Kane JM, Leucht S, Correll CU. Tardive dyskinesia risk with first- and second-generation antipsychotics in comparative randomized controlled trials: a meta-analysis. *World Psychiatry*. 2018;17:330-340. 10.1002/wps.20579 30192088 PMC6127753

[82] Li L, Le TH, Kim WS, et al. Predictors of relapse after discontinuing antipsychotics in patients with schizophrenia spectrum disorders. *Schizophrenia (Heidelb)*. 2025;11:42. 10.1038/s41537-025-00592-3 40082469 PMC11906741

[83] McCutcheon R, Beck K, Bloomfield MA, Marques TR, Rogdaki M, Howes OD. Treatment resistant or resistant to treatment? Antipsychotic plasma levels in patients with poorly controlled psychotic symptoms. *J Psychopharmacol*. 2015;29:892-897. 10.1177/0269881115576688 25788157 PMC4902121

[84] Crowley P, Healy J, McNamara N, et al. Duration of active psychosis: a scoping review of definition, measurement, and relationship with outcomes. *Schizophr Res*. 2025;285:232-241. 10.1016/j.schres.2025.09.032 41061565

